# Effect of Mn Addition on the Mechanical Properties and Ferroelectric Behavior of Bi_0.5_Na_0.5_TiO_3_ and 94(Bi_0.5_Na_0.5_TiO_3_)–6(BaTiO_3_) Ceramics

**DOI:** 10.3390/ma19061092

**Published:** 2026-03-12

**Authors:** Adriana Gallegos-Melgar, Jan Mayen, Maricruz Hernandez-Hernandez

**Affiliations:** 1 SECIHTI-InnovaBienestar de México, S.A.P.I. de C.V., Saltillo C.P. 25290, Mexico; maricruz.hdz@innovabienestar.mx; 2 SECIHTI-CIATEQ, A. C. San Luis Potosí, San Luis Potosí C.P. 78395, Mexico; jan.mayen@ciateq.mx

**Keywords:** lead-free ferroelectric ceramics, Bi_0.5_Na_0.5_TiO_3_ (BNT), BNT–BT, Mn doping, ferroelectric hysteresis, dielectric properties, nanoindentation

## Abstract

**Highlights:**

**What are the main findings?**

- Mn doping preserves single-phase perovskite structure in BNT and BNT-BT ceramics.
- 0.5 mol% Mn maximizes remanent polarization in BNT-BT (~33–34 μC/cm^2^).
- High Mn content causes grain coarsening and reduced densification.

**What are the implications of the main findings?**

- Low Mn levels enable tuning of ferroelectric response in lead-free BNT-BT ceramics.
- Excess Mn degrades electrical performance due to porosity and defect saturation.
- Mn-doped BNT-BT ceramics are promising for lead-free piezoelectric applications.

**Abstract:**

The effect of Mn addition on the structural, dielectric, ferroelectric, and mechanical properties of Bi_0.5_Na_0.5_TiO_3_ (BNT) and 0.94(Bi_0.5_Na_0.5_TiO_3_)–0.06(BaTiO_3_) (BNT–BT) ceramics was systematically investigated under identical processing conditions. Powders were calcined at 750 °C for 2 h and 900 °C for 2 h, followed by sintering at 1060 °C for 5 h. Mn contents of 0.5 and 5 mol% were selected to represent low-level substitution and near-saturation regimes. XRD confirmed single-phase perovskite formation within laboratory detection limits, while Raman spectroscopy revealed Mn-induced lattice distortions. Low Mn addition (0.5 mol%) enhanced densification and improved remanent polarization in BNT–BT (Pr = 33.5 μC/cm^2^). In contrast, 5 mol% Mn promoted grain coarsening, increased porosity, and reduced functional performance. Mechanical properties evaluated using two-parameter Weibull statistics showed composition-dependent variations in characteristic hardness and elastic modulus. The results demonstrate that Mn-doping effects depend strongly on both dopant concentration and host-lattice structural state, distinguishing beneficial substitution from defect-saturation behavior in lead-free BNT-based ceramics.

## 1. Introduction

Ferroelectric materials exhibit piezoelectric behavior and are widely used in capacitors, actuators, sensors, medical ultrasound devices, sonar systems, and energy harvesting applications [1,2,3,4]. Lead zirconate titanate (PZT) remains the benchmark piezoelectric ceramic due to its superior dielectric and electromechanical properties, particularly near its morphotropic phase boundary (MPB), where remanent polarization values of 45–50 μC/cm^2^ and coercive fields of approximately 10–12 kV/cm are typically reported [5,6]. However, increasing environmental concerns and regulatory restrictions related to lead toxicity have positioned lead-free ferroelectric materials as a priority direction in contemporary materials research [7].

Among the most studied lead-free systems are bismuth sodium titanate (Bi_0.5_Na_0.5_TiO_3_, BNT), bismuth potassium titanate (BKT), barium titanate (BT), potassium sodium niobate (KNN), and their solid solutions [8,9,10,11,12,13,14,15,16,17,18,19,20,21,22,23]. Despite considerable progress, most lead-free ceramics still underperform relative to PZT, particularly in terms of electromechanical coupling and polarization stability. One of the most effective strategies to enhance functional properties in lead-free ferroelectrics is the exploitation of MPB compositions, where structural instability and phase coexistence facilitate domain switching and enhanced polarization response [23,24,25,26].

In this context, the BNT–BT solid solution has attracted significant attention. Takenaka et al. identified an MPB region near 0.94(Bi_0.5_Na_0.5_TiO_3_)–0.06(BaTiO_3_), where enhanced dielectric, ferroelectric, and piezoelectric properties are achieved due to the coexistence of rhombohedral and tetragonal phases [27]. Beyond compositional tuning, chemical doping has been widely employed to further tailor the electrical response of BNT-based ceramics. Donor dopants (e.g., Nb^5+^) generally soften ferroelectric behavior by reducing coercive field and enhancing polarization, whereas acceptor dopants (e.g., Mn^2+^/Mn^3+^) tend to harden the lattice through defect dipole formation and domain-wall pinning [28,29,30].

Manganese incorporation has received particular attention in BNT- and BNT–BT-based ceramics due to its role in defect engineering; however, the interplay between dopant concentration, processing conditions, and resulting functional properties is not yet fully understood [28,29,31]. However, most previous studies focus on a single host composition—either BNT or BNT–BT—making it difficult to isolate matrix-dependent effects from dopant-induced phenomena. Recent studies have emphasized that the functional response of Mn-doped BNT-based ceramics is highly sensitive to dopant concentration, defect chemistry, and processing conditions. In particular, excessive Mn addition has been associated with increased dielectric loss, defect clustering, and degradation of ferroelectric stability, whereas low-level Mn doping can enhance domain-wall mobility and polarization response when properly controlled [32,33]. Moreover, comparative studies systematically evaluating Mn substitution in both matrices under identical synthesis and sintering conditions remain scarce. This limitation hinders a clear understanding of how the intrinsic structural state of the host lattice—BNT, widely described as a relaxor ferroelectric exhibiting non-ergodic behavior below its depolarization temperature, versus MPB-engineered BNT–BT with enhanced phase coexistence—governs the role of Mn-related defects, lattice distortion, and microstructural evolution. In addition to functional properties, the mechanical behavior of BNT-based ferroelectrics is a critical but relatively underexplored aspect, particularly in relation to dopant-induced microstructural changes. Previous studies have shown that appropriate dopant additions can enhance hardness, elastic modulus, and fracture toughness in BNT- and BNKT-based ceramics, while excessive dopant levels often promote abnormal grain growth and porosity, leading to mechanical degradation [10,34,35,36,37,38].

Therefore, the present work aims to provide a direct and systematic comparison of Mn-doped BNT and Mn-doped BNT–BT ceramics processed under identical conditions. By correlating structural evolution (lattice parameters and Raman-active modes), microstructural features (grain size and porosity), and multifunctional properties (dielectric, ferroelectric, and mechanical responses), this study clarifies the matrix-dependent role of Mn in lead-free perovskite ceramics. Particular attention is given to distinguishing beneficial low-level Mn doping from dopant saturation effects at high concentrations, thereby defining practical composition windows for multifunctional optimization. In addition to its role as a functional dopant in bulk ceramics, a high Mn concentration (5 mol%) was deliberately included in this study due to its relevance in the preparation of ceramic targets for pulsed laser deposition (PLD). In a previous work, Mn-modified BNT-based ceramics were successfully employed as dense and compositionally stable PLD targets for the growth of ferroelectric thin films, where target density, stoichiometric transfer, and ablation behavior play a critical role in film quality [39]. Accordingly, the 5 mol% Mn composition was selected not only to explore dopant saturation and defect-related effects in bulk ceramics, but also to establish a direct link with processing conditions relevant to epitaxial thin-film fabrication.

## 2. Materials and Methods

### 2.1. Sample Preparation

BNT and BNT–BT ceramics were prepared by solid-state reaction assisted by high-power milling. The precursor powders Bi_2_O_3_ (99.9%), Na_2_CO_3_ (99.9%), TiO_2_ (99.9%), and BaTiO_3_ (99.98%) (all from Sigma-Aldrich (St. Louis, MO, USA)) were weighed in stoichiometric proportions. Mn was introduced (as MnO_2_) at 0.5 and 5 mol% relative to the perovskite formula, producing the compositions listed in Table 1.

The mixed powders were calcined in two steps: 750 °C for 2 h, followed immediately by 900 °C for 2 h. After calcination, powders were re-milled and pressed into 10 mm diameter pellets. Sintering was performed at 1060 °C for 5 h in ambient atmosphere. Bulk density was measured by the Archimedes method; at least five pellets per composition were used to obtain representative averages.

### 2.2. Structural, Microstructural, and Spectroscopic Characterization

X-ray diffraction (XRD) patterns were collected from 20° to 80° (2θ) using a Rigaku Dmax2100 diffractometer (Rigaku Corporation, Tokyo, Japan) in Bragg–Brentano geometry with Cu Kα radiation (λ = 1.5406 Å). Phase identification was performed by indexing the diffraction peaks to the perovskite structure and by comparison with reference patterns. Within the detection limits of laboratory XRD, no secondary phases were detected. Raman spectra were obtained using a Dilor Labram II micro-Raman spectrometer (Horiba Jobin Yvon, France) equipped with a 514.5 nm Ar^+^ laser, operating in the 52–1000 cm^−1^ spectral range.

Microstructural observations were carried out using a JEOL EPMA JXA-8530F scanning electron microscope (JEOL Ltd., Tokyo, Japan) operated at an accelerating voltage of 10 kV, fractured surfaces were examined to evaluate intrinsic grain morphology and porosity.

### 2.3. Electrical and Ferroelectric Measurements

Ferroelectric hysteresis loops (P–E) were measured using a Radiant Technologies Precision Premier II ferroelectric tester (Radiant Technologies Inc., Albuquerque, NM, USA) coupled to a Trek 609E-6 high-voltage amplifier (Trek Inc., Lockport, NY, USA). Ferroelectric hysteresis loops were measured at room temperature using a triangular waveform at 1 kHz. The selected measurement frequency (1 kHz) represents a compromise between minimizing leakage contributions and maintaining consistent comparison with the literature reports measured under similar conditions. All measurements were performed on polished pellets with a final thickness of approximately 200 µm, allowing a maximum applied electric field of ~200 kV/cm. Dielectric properties were evaluated using an Agilent 4294A Precision Impedance Analyzer (Agilent Technologies, Santa Clara, CA, USA). Silver electrodes were applied to both faces of the sintered pellets using conductive silver paste. The electrode-coated samples were dried prior to testing. The average specimen thickness was ~200 μm. No electrical poling treatment was performed before the measurements.

### 2.4. Mechanical Properties

Mechanical properties were evaluated by Vickers microhardness testing and instrumented nanoindentation. Vickers hardness (HV) and fracture toughness *KIC* were determined from Vickers indentations, while nanoindentation was used to obtain hardness and the reduced elastic modulus E*. Vickers microhardness measurements were performed using a HV-1000 micro-Vickers hardness tester (Shanghai Lianer Testing Equipment Co., Ltd., Shanghai, China). Indentations were carried out under an applied load of 500 gf with a dwell time of 10 s. A minimum of 25 indentations per composition was performed to ensure statistical reliability.

Nanoindentation experiments were conducted using a Hysitron UBI 1 nanoindenter (Hysitron Inc., Minneapolis, MN, USA) equipped with a Berkovich diamond tip. Indentations were performed under load-control mode with a maximum load of 10 mN, loading/unloading rate of 10 mN/s. The reduced elastic modulus (E*) was calculated using the Oliver–Pharr method.

The Vickers hardness and elastic modulus values reported correspond to the characteristic parameters (HV0 and E0) obtained from a two-parameter Weibull statistical analysis.

The cumulative probability of failure was calculated using the median rank estimator, and the data were fitted using the linearized form of the Weibull distribution. The Weibull modulus (m) was obtained from the slope of the linear regression, while the characteristic values were derived from the intercept. This statistical treatment allows a robust assessment of the mechanical reproducibility of brittle ceramic systems, where local microstructural heterogeneity may influence indentation response.

## 3. Results

### 3.1. Phase Formation and Lattice Parameters

**Figure 1:**
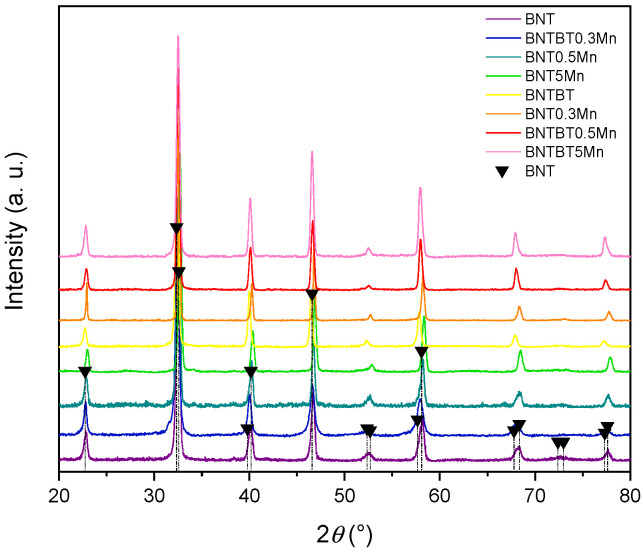
X-ray diffraction (XRD) patterns of BNT and BNT–BT ceramics with different Mn contents recorded in the 2θ range of 20–80°. All compositions exhibit a single-phase perovskite structure without detectable secondary phases or pyrochlore. The diffraction peaks can be indexed to the rhombohedral BNT-based perovskite structure, indicating that Mn addition does not alter the crystal symmetry within the detection limit of XRD. Small peak shifts observed with increasing Mn content suggest lattice parameter variations associated with Mn incorporation into the perovskite lattice.

X-ray diffraction patterns of undoped and Mn-doped BNT and BNT–BT ceramics (Figure 1) confirm the formation of single-phase perovskite structures within the detection limits of laboratory XRD. No secondary phases associated with Mn-rich oxides were detected, even at the highest Mn content (5 mol%), indicating effective incorporation of Mn into the perovskite lattice. However, systematic changes in peak positions and peak broadening were observed with increasing Mn content, suggesting lattice distortion induced by Mn substitution. For both BNT and BNT–BT matrices, low Mn content leads to slight lattice contraction, consistent with substitution at the Ti-site. At higher Mn content (5 mol%), deviations from linear lattice evolution are observed, indicating dopant saturation effects and increased structural disorder. These changes are more pronounced in the BNT–BT system, which is located near the morphotropic phase boundary (MPB) and is therefore more sensitive to compositional and defect-induced perturbations.

**Figure 2:**
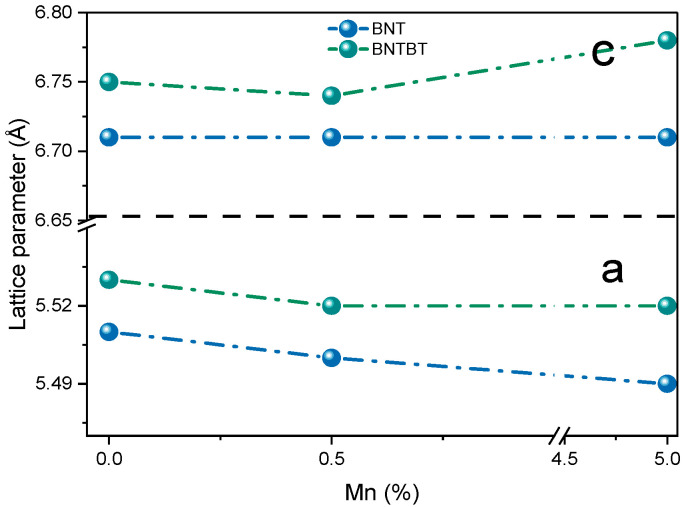
Evolution of lattice parameters as a function of Mn content for BNT and BNT–BT ceramics, estimated from peak-position analysis of XRD patterns: lattice parameter *a* and lattice parameter *c*.

For BNT-based ceramics (Figure 2), the lattice parameter *a* decreased with increasing Mn content, while c remained nearly constant. This behavior is consistent with partial substitution of Ti^4+^ (0.605 Å) by smaller Mn^4+^ ions (0.53 Å) [40]. For BNT–BT ceramics, both lattice parameters decreased at low Mn content, whereas at 5 mol% Mn, the *c* parameter increased, suggesting saturation effects and defect-driven lattice distortion.

### 3.2. Density and Microstructure

**Figure 3:**
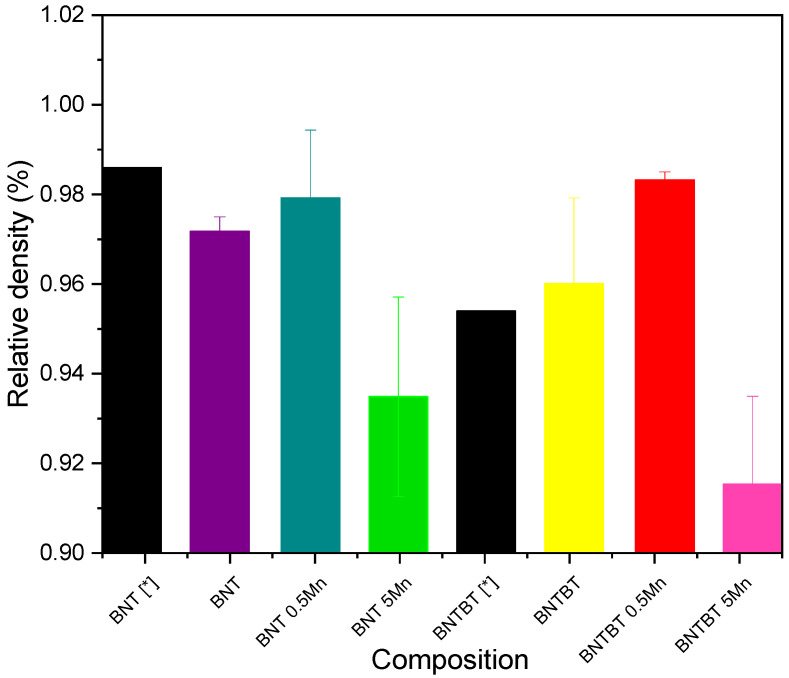
Density of BNT-based ceramics. (BNT–BT *) [43] (BNT *) [44].

The average bulk densities are presented in Figure 3. The highest relative densities within each matrix were obtained at 0.5 mol% Mn addition, reaching ~98%, whereas further Mn increase to 5 mol% resulted in densification deterioration [41,42].

### 3.3. Microstructural Characterization

**Figure 4:**
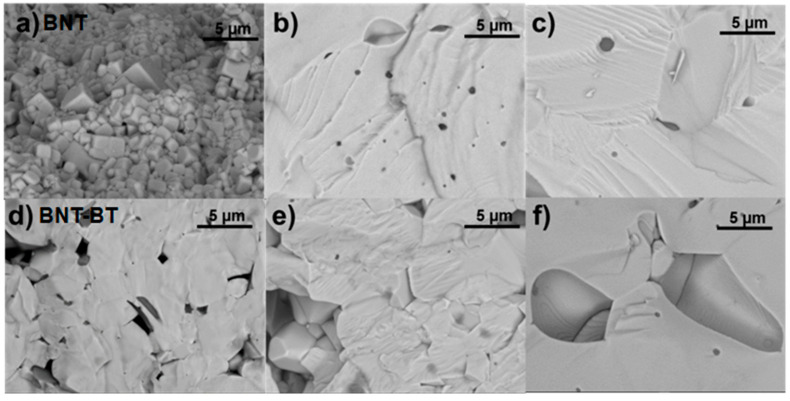

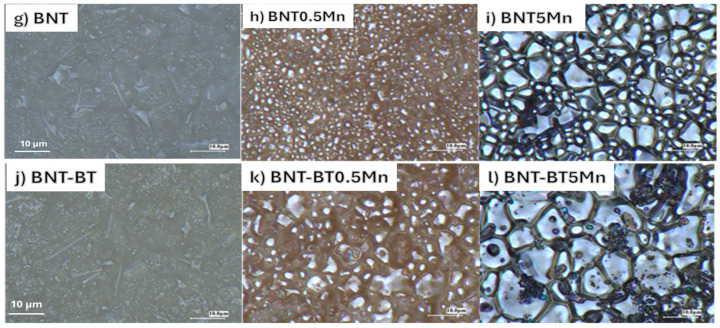
Microstructural characterization of BNT and BNT–BT ceramics with different Mn contents. (**a**–**f**) SEM micrographs of fractured surfaces of BNT, BNT0.5Mn, BNT5Mn, BNT–BT, BNT–BT0.5Mn, and BNT–BT5Mn ceramics (scale bar: 10 µm). (**g**–**i**) Optical micrographs of BNT ceramics with increasing Mn content (scale bar: 10 µm). (**j**–**l**) Optical micrographs of BNT-BT ceramics with increasing Mn content (scale bar: 10 µm). Both SEM and optical microscopy reveal a progressive Mn-induced grain growth and increased porosity at high Mn contents.

Figure 4 summarizes the microstructural features of BNT and BNT–BT ceramics with different Mn contents, as observed by scanning electron microscopy (SEM) and optical microscopy. The combined use of both techniques allows a comprehensive evaluation of grain size evolution and porosity development as a function of Mn concentration.

#### 3.3.1. SEM Observations

SEM micrographs of fractured surfaces (Figure 4a–f) reveal that undoped BNT ceramics exhibit a fine-grained microstructure composed of submicrometric equiaxed grains with limited porosity, indicating effective densification. With Mn addition, a progressive grain growth is observed in the BNT system. At low Mn contents, grain coarsening remains moderate, while at 5 mol% Mn, grains reach several micrometers in size and coexist with enlarged pores located mainly at grain boundaries. This microstructural evolution is consistent with the reduction in bulk density measured for highly doped BNT ceramics.

A similar trend is observed for the BNT–BT system. Undoped BNT–BT presents fine grains with relatively homogeneous size distribution, whereas Mn-doped samples show increasingly heterogeneous microstructures. In particular, BNT–BT ceramics doped with 5 mol% Mn display pronounced abnormal grain growth, characterized by large polygonal grains and significant residual porosity. This microstructural degradation correlates with the lower densification and degraded ferroelectric and dielectric properties observed at high Mn contents.

#### 3.3.2. Optical Microscopy Observations

Optical micrographs of polished surfaces for BNT and BNT–BT ceramics (Figure 4g–l) further illustrate the grain growth behavior induced by Mn addition. In undoped BNT, the apparent grain size is close to the resolution limit of optical microscopy, confirming a submicrometric microstructure. The addition of Mn leads to a clear increase in apparent grain size, reaching approximately ~2.0 µm for 0.5 mol % Mn and ~5.5 µm for 5 mol % Mn. At the highest Mn concentration, grains become large, polygonal, and well defined.

For the BNT–BT system, optical microscopy reveals an even more pronounced grain growth. Undoped BNT–BT shows fine grains with an apparent size of ~1.2 µm, while Mn-doped samples exhibit progressively larger grains, reaching ~3.2 µm at 0.5 mol % Mn and ~6.8 µm at 5 mol % Mn. The presence of large grains and enhanced contrast at grain boundaries in highly doped samples suggests increased grain boundary grooving and porosity, in agreement with SEM observations.

#### 3.3.3. Consistency Between SEM and Optical Microscopy Results

The microstructural features observed by SEM and optical microscopy are fully consistent and complementary. SEM provides high-resolution evidence of grain morphology, submicrometric grain size, and porosity evolution, particularly in undoped and lightly doped ceramics where grain dimensions approach the resolution limit of optical microscopy. In contrast, optical microscopy captures the overall grain morphology and size distribution in moderately and highly doped samples, where grain sizes exceed several micrometers.

In both BNT and BNT–BT systems, Mn addition promotes grain growth, with a clear transition from fine and relatively homogeneous microstructures at low Mn contents to coarse and heterogeneous microstructures at 5 mol % Mn. The agreement between both techniques confirms that the apparent grain sizes estimated from optical microscopy reflect the same Mn-induced grain coarsening process revealed by SEM. Moreover, the combined analysis demonstrates that excessive Mn content leads to abnormal grain growth and increased porosity, which explains the observed reduction in densification and the deterioration of electrical properties at high Mn concentrations.

#### 3.3.4. Raman Spectroscopy

Raman spectroscopy provides further insight into local structural distortions. The Raman spectra of Mn-doped samples exhibit noticeable shifts and broadening of modes associated with TiO_6_ octahedral vibrations, particularly in the mid-frequency region. In BNT–BT ceramics, Mn addition enhances mode asymmetry and band overlap, consistent with increased phase coexistence and local symmetry breaking near the MPB. These spectroscopic changes become more pronounced at higher Mn concentrations, reflecting increased lattice distortion and defect-related disorder.

**Figure 5:**
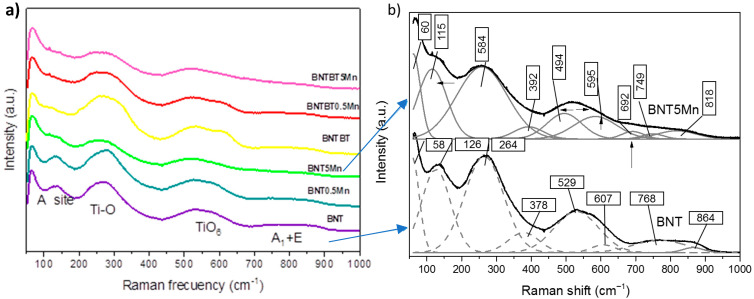
(**a**) Raman spectra of BNT and BNT–BT-based ceramics with different Mn contents recorded at room temperature. (**b**) The spectra were deconvoluted to identify the main vibrational modes associated with A–O, Ti–O, and TiO_6_ octahedral vibrations.

Raman spectra for all compositions are shown in Figure 5, and the identified vibrational modes are summarized in Table 2. The observed modes are consistent with previous reports for BNT [45,46,47,48,49] and BNT–BT systems [50,51]. Compared to BNT, BNT–BT exhibits shift toward lower frequencies, indicating reduced distortion and increased tetragonal symmetry associated with the MPB region.

Mn doping leads to systematic changes in the high-wavenumber region (600–800 cm^−1^), including peak narrowing and downshifts, suggesting increased lattice rigidity and modified oxygen-vacancy concentration. In samples doped with 5 mol % Mn, an additional mode near ~690 cm^−1^ appears, which can be attributed to Mn–O_6_ vibrations and indicates Mn substitution at Ti sites, as reported for other perovskite systems [50,52,53].

**Table 2:** Raman identified vibrational modes.

Composition	A-O	Na-O Ba-O	Ti-O	Ti-O	TiO_6_	TiO_6_	TiO_6_	TiO_6_	MnO_6_
BNT [52]	--	146	249	281–318	542	--	--	812	--
BNT-xBT, x < 6 [54]	--	146	249	281	542	--	--	812	--
BNT	58	126	264	378	529	607	768	864	--
BNT0.5Mn	59	124	270	393	532	593	738	834	--
BNT5Mn	60	115	258	392	494	495	749	818	692
BNT–BT	63	99	269	322	533	619	767	857	--
BNT–BT0.5Mn	63	94	235	269	526	614	770	854	--
BNT–BT5Mn	61	106	252	356	519	620	751	793	695

Undoped BNT and BNT–BT ceramics exhibit characteristic modes in the low-frequency region (~60–150 cm^−1^), intermediate region (~250–400 cm^−1^), and high-frequency region (~500–800 cm^−1^), consistent with rhombohedral perovskite structures. With Mn addition, systematic shifts and peak narrowing are observed in the high-wavenumber region, indicating increased lattice rigidity and modified oxygen-vacancy concentration. At high Mn content (5 mol%), an additional Raman mode appears near ~690 cm^−1^, which is attributed to Mn–O_6_ octahedral vibrations, confirming Mn incorporation into the perovskite lattice.

### 3.4. Ferroelectric and Dielectric Properties

**Figure 6:**
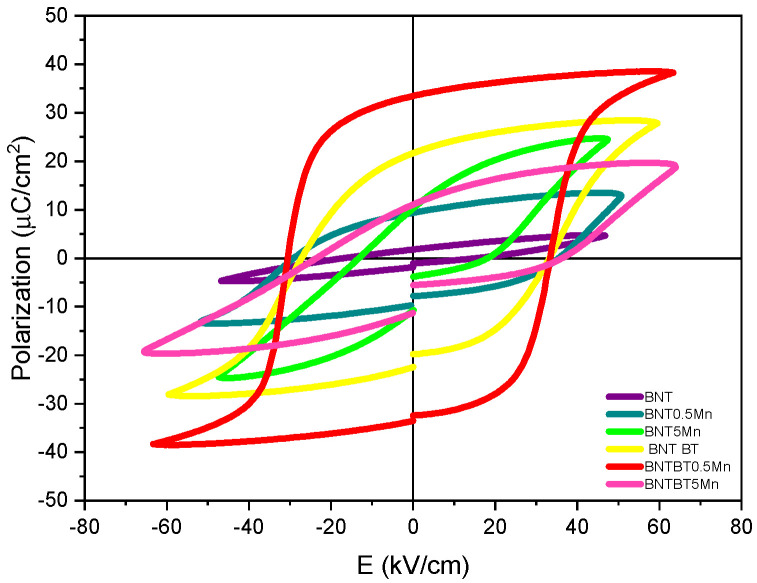
Polarization–electric field (*P*–*E*) hysteresis loops of BNT and BNT–BT ceramics with different Mn contents measured at room temperature. The electric field was increased until hysteresis loop saturation or dielectric breakdown occurred; for clarity, the loops are displayed within the electric-field range where stable switching behavior was observed.

Ferroelectric hysteresis loops are shown in Figure 6, and the extracted parameters are summarized in Table 3. During ferroelectric measurements, the electric field was progressively increased until either dielectric breakdown occurred or the hysteresis loop approached saturation. Since the dielectric strength varied among compositions, the effective maximum electric field differed slightly between samples. In most cases, the hysteresis loops reached stable switching behavior within the electric-field range displayed in Figure 6; therefore, the loops are shown within this range to facilitate comparison among compositions. All samples exhibit ferroelectric behavior, although the loop shape and switching characteristics strongly depend on Mn content and the host matrix. For P–E measurements, the sintered pellets (~500 µm) were polished to an average thickness of ~200 µm, allowing the application of a maximum electric field of approximately 200 kV/cm (4 kV maximum voltage). Differences in *Pr* values for undoped BNT are attributed to processing route, sintering profile, and sample thickness, which significantly influence leakage and switching behavior.

Mn addition significantly influences the remanent polarization (Pr) and coercive field (Ec). In the BNT system, low Mn incorporation modifies the switching behavior, whereas Mn incorporation initially increases the coercive field at 0.5 mol%, followed by a reduction at 5 mol%, suggesting a transition from defect-induced hardening to conduction-assisted switching at higher Mn concentration. In contrast, BNT–BT ceramics doped with 0.5 mol% Mn exhibit the highest remanent polarization (Pr = 33.5 µC/cm^2^), while excessive Mn content (5 mol%) results in reduced polarization, consistent with microstructural degradation, increased defect density, and reduced densification at higher dopant levels.

To provide quantitative context, Table 3 compares the Pr and Ec values obtained in this work with recent literature reports on BNT and BNT–BT systems [55,56]. The comparison explicitly considers the maximum applied electric field (Emax), since hysteresis-loop magnitude is field-dependent. The Pr and Ec values measured for BNT–BT compositions fall within the ranges reported for MPB-engineered systems, confirming the physical consistency of the present results. Differences observed for BNT-based compositions are attributed to defect-mediated domain-wall pinning and leakage contributions associated with Mn-related defect dipoles and oxygen vacancies.

**Figure 7:**
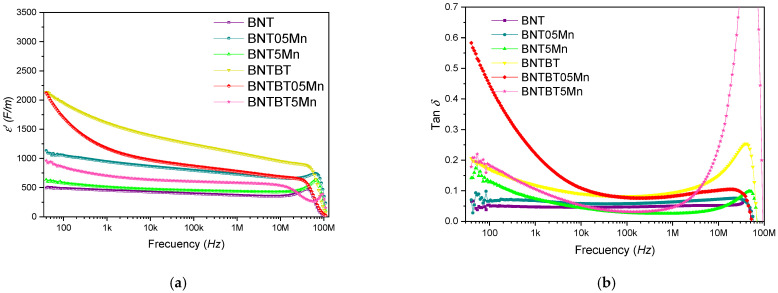
Frequency dependence of the dielectric properties of BNT and BNT–BT ceramics with different Mn contents measured at room temperature using silver electrodes in the frequency range from 10^1^ to 10^7^ Hz: (**a**) real part of the dielectric permittivity (*ε*′) and (**b**) dielectric loss (tan *δ*).

Dielectric permittivity and loss as a function of frequency are shown in Figure 7. In BNT ceramics, 0.5 mol% Mn increased the dielectric constant from 456 to 948 at 1 kHz, suggesting enhanced polarization contributions at low dopant levels. In contrast, in BNT–BT ceramics, the dielectric constant decreases with increasing Mn content, reflecting the complex role of Mn, which may act predominantly as a donor dopant in BNT but exhibits mixed donor/acceptor behavior in BNT–BT, potentially involving substitution at multiple lattice sites (Ti^4+^, Ba^2+^, Bi^3+^, and Na^+^) [57].

The polarization–electric field (P–E) behavior, therefore, reflects a balance between structural distortion and defect chemistry. Although Mn incorporation induces lattice distortion—particularly in BNT–BT compositions near the morphotropic phase boundary—this structural modification does not lead to a monotonic increase in remanent polarization at higher Mn contents. At low Mn levels, local modification of the TiO_6_ octahedral environment may facilitate polarization switching. However, at higher concentrations, defect accumulation, residual porosity, and microstructural heterogeneity restrict domain-wall mobility, resulting in defect-dominated switching behavior.

Consequently, the observed loop shapes and coercive field values are consistent with defect-mediated domain-wall pinning rather than anomalous ferroelectric behavior and align with the quantitative comparison presented in Table 3.

**Table 3:** Quantitative comparison of ferroelectric parameters with recent literature.

System	Mn (mol%)	Pr (µC/cm^2^)	E_c_ (kV/cm)	E_max_ (kV/cm)	Reference
BNT	0	29	61.1	>100	[56]
BNT	0	1.8	17.7	200	This work
BNT	0.5	9.3	35.6	200	This work
BNT	5	10.1	18.5	200	This work
BNT–6BT	0	21–30 *	25–40 *	>100	[55]
BNT–6BT	0	21.7	33	200	This work
BNT–6BT	0.5	33.5	33	200	This work
BNT–6BT	5	11.2	36.5	200	This work

* Correspond to measurements reported within a range of experimental conditions in the cited literature.

In the dielectric diagrams (Figure 7), all compositions show a typical decrease in ε′ with increasing frequency, associated with reduced space–charge and dipolar polarization contributions at high frequencies. Mn addition modifies both ε′ and tan δ, enhancing the dielectric permittivity in BNT ceramics at low Mn content, while in BNT–BT ceramics excessive Mn leads to reduced ε′ and increased dielectric losses at high frequencies, consistent with the microstructural degradation and increased porosity observed at high Mn concentrations.

### 3.5. Mechanical Properties

Mechanical properties obtained by nanoindentation and Vickers microhardness are summarized in Table 4, the hardness values reported, and the reduced elastic modulus derived from a two-parameter Weibull distribution. Weibull modulus values above 10 indicate acceptable statistical reliability of the indentation dataset. While microstructural heterogeneity is observed at high Mn contents, the dispersion of indentation data remained limited, validating the use of characteristic parameters for comparative purposes. Therefore, the characteristic hardness and elastic modulus values obtained from the two-parameter Weibull analysis were considered statistically representative of each composition and were used for comparative evaluation in the discussion. The influence of Mn addition on hardness and elastic modulus is evident for both BNT and BNT–BT systems. Similar trends have been reported for BNT- and BNKT-based ceramics, where appropriate dopant additions promote grain growth control and local stiffening, resulting in enhanced hardness and elastic modulus values [36,38,58].

In the present work, ceramics doped with moderate Mn content (0.5 mol%) exhibit improved mechanical performance, which can be associated with enhanced densification and a more homogeneous microstructure. Comparable improvements in hardness and elastic modulus due to dopant-induced microstructural refinement have been reported in Zr-, La-, and Nb-modified BNT-based systems [35,36,38,58].

In contrast, excessive Mn addition (5 mol%) leads to increased porosity and grain coarsening, which adversely affects macroscopic mechanical and electrical properties. This behavior is consistent with previous studies reporting that high dopant concentrations can deteriorate densification and promote defect accumulation in perovskite ceramics [41,42]. Despite this, nanoindentation results indicate locally increased stiffness, suggesting that Mn incorporation may strengthen individual grains even when the overall mechanical integrity is compromised by porosity.

## 4. Discussion

The results obtained in this study demonstrate that manganese plays a multifaceted role in BNT- and BNT–BT-based ferroelectric ceramics, strongly influencing structural, microstructural, electrical, and mechanical behavior as a function of dopant concentration and host composition.

### 4.1. Structural Incorporation and Lattice Distortion

From a structural perspective, the evolution of lattice parameters with Mn content indicates that Mn is incorporated into the perovskite lattice, primarily substituting Ti^4+^ at the B-site. In Mn-doped BNT, the reduction in the lattice parameter a is consistent with the smaller ionic radius of Mn^4+^ compared to Ti^4+^, as reported by Shannon [40]. In contrast, BNT–BT ceramics exhibit a non-monotonic evolution of lattice parameters, particularly an increase in the c parameter at 5 mol% Mn. This behavior suggests dopant saturation and defect-induced lattice distortion, which has been associated with altered tetragonality and enhanced phase coexistence near the morphotropic phase boundary (MPB) [27,51].

Raman spectroscopy provides complementary insight into these local structural modifications. The shift in vibrational modes toward lower frequencies in BNT–BT relative to BNT reflects reduced lattice distortion and increased tetragonal symmetry, consistent with MPB-related structural instability [50,51]. Furthermore, the appearance of an additional Raman mode near ~690 cm^−1^ in samples containing 5 mol% Mn is attributed to Mn–O_6_ octahedral vibrations, supporting Mn incorporation at the Ti site, as previously reported for Mn-modified perovskite systems [50,52,53].

### 4.2. Densification and Microstructural Evolution

The densification behavior highlights the concentration-dependent effect of Mn. Low Mn addition (0.5 mol%) promotes densification in both BNT and BNT–BT ceramics, whereas higher Mn content (5 mol%) results in reduced density. Similar trends have been reported in Mn-doped oxide ceramics, where excessive dopant levels hinder mass transport and promote defect accumulation during sintering [41,42]. In BNT–BT ceramics, this effect is further aggravated by the volatility of Na and Bi during high-temperature sintering, which facilitates pore formation and entrapment [59,60].

Microstructural observations corroborate these findings. Excessive Mn addition promotes abnormal grain growth and increased porosity, indicating that Mn acts as a sintering aid only within a limited compositional window. Although MnO_2_ can promote transient liquid-phase sintering, its decomposition and associated oxygen release at elevated temperatures may intensify A-site volatilization, thereby deteriorating the final microstructure when present in excess [41,42].

### 4.3. Ferroelectric and Dielectric Response

The ferroelectric and dielectric properties are closely coupled to the observed structural and microstructural evolution. In BNT ceramics, Mn incorporation significantly influences polarization switching behavior. At 0.5 mol%, the coercive field increases from 17.7 kV/cm (undoped BNT) to 35.6 kV/cm, indicating defect-induced domain-wall pinning and a hardening effect. At 5 mol% Mn, the coercive field decreases to 18.5 kV/cm, approaching the undoped value, which suggests that excessive dopant concentration promotes defect saturation and enhanced leakage contributions, partially facilitating polarization reversal. These results indicate a non-monotonic evolution of switching behavior governed by the competition between defect-mediated hardening and conduction-assisted domain-wall motion [57].

In BNT–BT ceramics, the role of Mn is more complex. While 0.5 mol% Mn significantly enhances remanent polarization, higher Mn content leads to reduced dielectric permittivity and polarization. This behavior suggests a transition from beneficial low-level substitution to defect-dominated behavior at higher Mn concentrations, potentially involving mixed donor–acceptor effects across multiple lattice sites (Ti^4+^, Ba^2+^, Bi^3+^, and Na^+^) [28,57]. The proximity of BNT–BT to the MPB amplifies the sensitivity of polarization mechanisms to Mn-induced lattice distortion and defect chemistry, enabling enhanced polarization at low Mn content but accelerating degradation once dopant saturation is reached.

Low-level Mn addition introduces controlled lattice distortions, as evidenced by subtle lattice-parameter variations and Raman mode shifts, which promote enhanced domain-wall mobility and polarization response. In contrast, high Mn content leads to dopant saturation, reflected by non-linear lattice evolution, Raman band broadening, and microstructural degradation. These features disrupt long-range ferroelectric order and enhance defect-related polarization mechanisms such as Maxwell–Wagner interfacial polarization.

Although such mechanisms may increase dielectric permittivity at low frequencies, they are accompanied by elevated dielectric loss. High dielectric loss is undesirable for functional applications, as it results in increased energy dissipation, thermal instability, and reduced efficiency in capacitor and actuator devices. Consequently, despite apparent permittivity enhancement, compositions with high loss values exhibit limited practical applicability. In this context, low Mn-doped compositions represent an optimal compromise between enhanced dielectric response and acceptable loss levels.

### 4.4. Mechanical Behavior

The mechanical properties follow trends consistent with the observed microstructural evolution. Moderate Mn addition improves hardness and elastic modulus due to enhanced densification and grain-boundary strengthening, in agreement with previous reports on doped BNT- and BNKT-based ceramics [10,36,38]. The differences observed between nanoindentation and Vickers hardness measurements can be attributed to the distinct length scales probed by each technique: nanoindentation reflects local mechanical behavior sensitive to Mn-induced lattice distortion and defect-related hardening, whereas Vickers hardness represents a macroscopic response integrating grain boundaries, residual porosity, and microstructural heterogeneity.

At higher Mn concentrations, locally increased stiffness detected by nanoindentation is offset by abnormal grain growth and increased porosity, which degrade the effective load-bearing capacity and overall mechanical integrity. Similar trade-offs between local strengthening and global mechanical performance have been reported in perovskite ceramics with high dopant concentrations [41]. It is important to note that nanoindentation probes local grain-level mechanical response, whereas macroscopic density reflects global structural integrity; therefore, local stiffness enhancement may coexist with reduced bulk densification.

### 4.5. Defect Chemistry and Overall Implications

The combined structural, electrical, and mechanical behavior of Mn-doped BNT and BNT–BT ceramics can be attributed to the ionic radius mismatch between Mn^3+^/Mn^4+^ ions and Ti^4+^ cations, which induces local lattice distortion and modifies TiO_6_ octahedral symmetry. Charge compensation is likely accommodated through oxygen vacancies and defect-dipole complexes. At low Mn concentrations, controlled lattice distortion and moderate defect density enhance functional responses, whereas excessive Mn content leads to defect saturation, restricted domain-wall mobility, increased dielectric loss, and degraded mechanical and electrical performance.

Overall, these results demonstrate that Mn acts as an effective dopant for tailoring the multifunctional properties of BNT and BNT–BT ceramics when used at low concentrations. However, excessive Mn addition introduces detrimental effects related to defect saturation, volatilization, and microstructural degradation. Careful control of Mn content is therefore essential to optimize the balance between structural stability, ferroelectric performance, and mechanical reliability in lead-free perovskite ceramics.

## 5. Conclusions

This study systematically evaluated the effect of Mn doping on the structural, microstructural, electrical, and mechanical properties of BNT and BNT–BT lead-free ferroelectric ceramics. The following conclusions can be drawn:

Mn is incorporated into the perovskite lattice primarily through partial substitution at the Ti^4+^ B-site, inducing lattice distortion that depends on both dopant concentration and host composition. Structural analysis reveals a monotonic lattice contraction in Mn-doped BNT, while BNT–BT exhibits non-linear lattice evolution associated with dopant saturation and MPB-related structural instability.

Low Mn addition (≤0.5 mol%) promotes densification and microstructural homogeneity in both systems, whereas excessive Mn content (5 mol%) leads to abnormal grain growth, increased porosity, and reduced bulk density, particularly in BNT–BT ceramics due to enhanced A-site volatilization.

Raman spectroscopy confirms Mn-induced local structural modification and supports partial B-site substitution, with the emergence of Mn–O_6_-related vibrational modes at high Mn content and enhanced phase coexistence in BNT–BT near the MPB.

Ferroelectric and dielectric properties are strongly dependent on Mn concentration. Low-level Mn doping enhances remanent polarization and dielectric response, especially in BNT–BT ceramics, while higher Mn content results in dopant saturation, increased dielectric loss, and degraded polarization behavior.

Mechanical properties reflect the underlying microstructural evolution. Moderate Mn addition improves hardness and elastic modulus due to enhanced densification and grain-boundary strengthening, whereas excessive Mn addition degrades macroscopic mechanical integrity despite locally increased stiffness.

The overall functional response results from a balance between beneficial lattice distortion at low Mn concentrations and defect-dominated behavior at higher dopant levels, governed by ionic radius mismatch and charge-compensation mechanisms involving oxygen vacancies.

In summary, controlled low-level Mn doping is an effective strategy for optimizing the multifunctional performance of BNT and BNT–BT ceramics, while excessive Mn addition leads to structural and functional degradation. These findings highlight the importance of precise dopant control in the design of high-performance, lead-free ferroelectric ceramics.

## Figures and Tables

**Table 1:** Nomenclature of the sample compositions in this research.

Composition	Nomenclature
Bi_0.5_Na_0.5_TiO_3_	BNT
Bi_0.5_Na_0.5_TiO_3_ + 0.5%mol MnO_2_	BNT0.5Mn
Bi_0.5_Na_0.5_TiO_3_ + 5%mol MnO_2_	BNT5Mn
0.94(Bi_0.5_Na_0.5_TiO_3_)-0.06(BaTiO_3_)	BNT–BT
0.94(Bi_0.5_Na_0.5_TiO_3_)-0.06(BaTiO_3_) + 0.5%mol MnO_2_	BNT–BT0.5Mn
0.94(Bi_0.5_Na_0.5_TiO_3_)-0.06(BaTiO_3_) + 5%mol MnO_2_	BNT–BT5Mn

**Table 4:** Summary of microstructural, densification, ferroelectric/dielectric, and mechanical parameters of BNT- and BNT–BT-based ceramics.

Composition	Avg. Grain Size (µm)	Relative Density (%)	Pr (µC/cm^2^)	tan δ at 1 kHz (%)	Characteristic Hardness *H*_0_ Obtained from Two-Parameter Weibull Analysis Vickers Hardness HV (GPa)	Characteristic Reduced Elastic Modulus *E*_0_ Obtained from Two-Parameter Weibull Analysis Nanoindentation E* (*b*)
BNT	<1.0	~97	1.8	4	3.18	60
BNT0.5Mn	~2.0	~98	9.3	6	6.21	96
BNT5Mn	~5.5	~93	10.1	7	3.00	98
BNT–BT	~1.2	~96	21.7	11	6.54	75
BNT–BT0.5Mn	~3.2	~98	33.5	21	5.90	94
BNT–BT5Mn	~6.8	~91	11.2	10	7.70	139

## Data Availability

The original contributions presented in this study are included in the article. Further inquiries can be directed to the corresponding author.
